# Explaining better hearing in Norway: a comparison of two cohorts 20 years apart - the HUNT study

**DOI:** 10.1186/s12889-021-10301-1

**Published:** 2021-01-28

**Authors:** Bo Engdahl, Hein Stigum, Lisa Aarhus

**Affiliations:** 1 Department of Chronic Diseases and Ageing, Norwegian Institute of Public Health, Postbox 4404 Nydalen, N-0403 Oslo, Norway; 2 Department of Occupational Medicine and Epidemiology, National Institute of Occupational Health, Oslo, Norway

**Keywords:** Causal inference, Ear infections, Hearing loss, Mediation analysis, Occupational noise, Smoking

## Abstract

**Background:**

The hearing function at a given age seems to have improved in more recent born cohorts in industrialized countries. But the reasons for the improvement have not yet been explained.

**Methods:**

We investigated the extent to which better hearing in Norway is attributed to modifiable risk factors by using representative demographic and audiometric data from two cohorts of the Trøndelag Health Study, HUNT2 (1996–1998) and HUNT4 (2017–2019). We estimated natural indirect effects using causal inference methods in order to assess whether cohort improvement in hearing thresholds (HTs) was mediated by occupational noise exposure, recurrent ear infections, smoking and education.

**Results:**

The improvement in HTs from HUNT2 to HUNT4 was 2.8 and 3.0 dB at low respectively high frequencies. Together all risk factors mediated this improvement by 0.8 dB (95% CI 0.7–0.9) and 0.8 dB (95% CI 0.7–0.9) respectively, corresponding to mediated proportions of 27 and 28%. Substantial mediation was specifically found for occupational noise in men and recurrent ear infections in women (mediated proportions of 11 and 17% at high frequencies, respectively).

**Conclusions:**

Increased education, less occupational noise exposure, ear infections and smoking contributed considerably to better hearing in Norway the last two decades.

**Supplementary Information:**

The online version contains supplementary material available at 10.1186/s12889-021-10301-1.

## Background

Sense organ diseases, among which hearing loss is the most common, was the leading cause of years lived with disability for elderly in 2015 [1]. The world’s population is aging rapidly, and unless action is taken, WHO estimates that the number of people with disabling hearing loss globally could rise from 466 million in 2018 to 630 million by 2030 and potentially to over 900 million in 2050 [2]. Fortunately, studies suggest that the hearing function at a given age has improved in more recent born cohorts in industrialized countries such as USA [3–5], Sweden [6] and Norway [7] although the trends for younger individuals show mixed results [8–12].

Several environmental and behavioral risk factors for hearing loss may have declined in the twentieth century potentially contributing to this generational improvement. Important risk factors for hearing loss are occupational noise exposure [13–16], recreational noise such as fire arms [16, 17], recurrent ear infections [18], and to some degree cardiovascular risk factors such as smoking, diabetes and hypertension [13, 14, 19, 20]. Hearing loss has also been linked to socioeconomic status and educational attainment [14, 21]. General improvements in socioeconomic status may be linked to reduced noise exposure, a healthier lifestyle and a general improvement in welfare and thus also better hearing health. To the best of our knowledge, only two studies have attempted to explain the generational improvement in hearing [22, 23]. While the first study ascribed some of the progress to an increase in educational attainment [22], its follow-up, assessing generational decrease in hearing loss incidence, failed to attribute the decrease to changes in any measured known risk factors such as cardiovascular factors, metabolic factors, and work-related noise [23]. Thus, the explanation for the generational differences remains unknown.

The aim of this paper is to examine to what extent the better hearing in recent birth cohorts is attributed to change in the modifiable risk factors for hearing loss occupational noise, ear infections and smoking. This by estimating natural indirect effects using causal inference methods.

## Methods

### Study sample

HUNT2 Hearing (1996–1998) and HUNT4 Hearing (2017–2019) were part of a large general health-screening study for the entire adult population of Nord-Trøndelag County (HUNT).

HUNT2 Hearing included 17 of the 24 municipalities in the county. The participation rate was 63%, and altogether, 51,529 persons attended. HUNT4 Hearing took part in the six larger municipalities, representing about two thirds of the county. The participation rate was 43%, and altogether, 28,388 persons attended. The hearing studies are described in detail elsewhere [7, 24]. After excluding persons with missing questionnaires or non-valid audiometry, the final cross-sectional samples comprised 49,594 and 26,606 participants in HUNT2 respectively HUNT4.

Longitudinal audiometric data were available for 12,115 subjects participating in both HUNT2 and HUNT4 hearing.

### Measurements

Detailed information about the measurements is described elsewhere [7, 24]. In short, both hearing studies included a questionnaire, otoscopy and pure-tone audiometry following the same automatic audiometric procedure. Pure-tone air-conduction hearing thresholds levels were determined in accordance with ISO 8253-1 [25], with fixed frequencies at the eight test frequencies 0.25–8 kHz. Hearing thresholds (HTs) were defined relative to the hearing threshold levels of the population of otologically normal subjects aged 19–23 years [7].

#### Outcome

We defined HTs averaged over both ears over the frequencies 0.5, 1 and 2 kHz (low frequency) and 3, 4 and 6 kHz (high frequency).

#### Mediators

To investigate factors that may explain the change in hearing, we selected three modifiable risk factors a priori which are known to be associated with hearing loss and to have been reduced in the population between the two study waves. These were self-reports of occupational noise (regularly been exposed to loud noise at your present or previous work, no/less than 5 h/week, 5–15 h/week, > 15 h/week); recurrent ear infections (no/maybe/yes), and daily smoking (never/former/current). In addition, we considered education (primary school/secondary school/university < 4 years/university>=4 years). Education was obtained from national registers and considered complete.

#### Sources of confounding and testable mediations

**Fig. 1:**
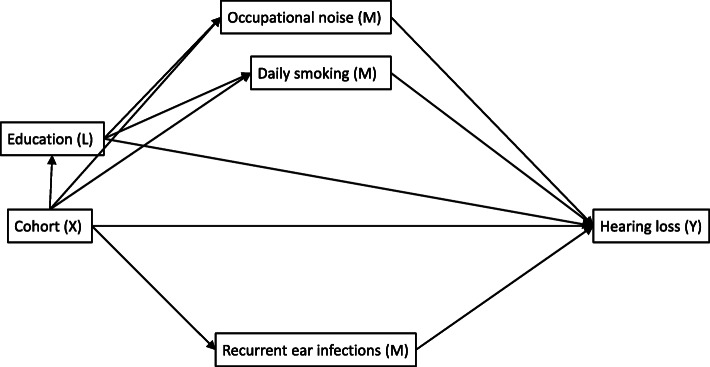
Direct acyclic graph (DAG) showing the assumed causal relationships between cohort (X) and hearing loss (Y) including a set of mediators (M) and intermediate mediator/confounder (L). The exogenous variables age and sex are omitted from the diagram but must be adjusted for in the analyses

We constructed a directed acyclic graph (DAG) using DAGitty software to identify variables that have a plausible, causal effect on the relationship between birth cohort and hearing loss (Fig. 1). The DAG implied mediation by recurrent ear infections to be testable after controlling for age and sex. Occupational noise and daily smoking required additional control for education, an intermediate confounder (a common cause of the mediator and the outcome which is also causally affected by the exposure).

### Statistical analyses

#### Concepts of mediation analyses

Concepts and methods of mediation analyses have changed over the last two decades [26]. Traditional mediation analysis estimates *controlled* direct (and indirect) effects. The controlled direct (and indirect) effects, found by comparing models with and without the mediator, are limited to linear models with no interaction between the exposure and the mediator. They also require no unmeasured confounding between exposure and outcome, and between mediator and outcome. The newer, causal inference methods estimate *natural* direct (and indirect) effects. The natural direct (and indirect) effects are more general and can be estimated also for non-linear models with or without exposure-mediator interaction. As for the controlled effects, they require no unmeasured confounding between exposure and outcome, and between mediator and outcome. In addition, the natural effects also require no unmeasured confounders between the exposure and the mediator. Lastly the natural effects require no confounders (unmeasured or measured) between the mediator and the outcome that are effects of the exposure (intermediate confounders). This excludes setting with multiple dependent mediators. Natural effects may still be estimated under such settings, but then under (parametric) assumptions of no exposure-intermediate confounder interaction and a linear effect of the exposure dependent confounder on the outcome [27]. Using a linear regression model for the outcome including the relevant non-linear and interaction terms makes it possible to test these assumptions and choose the correct identifying assumptions to estimate the natural direct and indirect effects [27].

#### Estimations in the present study

We estimated natural indirect effects by different causal inference methods. First, we estimated the effects of all mediators together. The effects of all mediators together do not require any special assumptions [28–30]. We fitted the joint mediated effect using imputation-based natural effect models of the R package “medflex” [29]. All exposure-mediation interaction terms were included (example code given in Online Resource 1, part A). We compared the effect estimates with estimates found by traditional analyses assuming no exposure-mediation interaction by regressing hearing thresholds on cohort and comparing two linear regression models - with and without the mediators.

Second, we estimated the specific natural indirect effects for each mediator. The effects for each mediator were estimated via parametric G-computation with Monte Carlo simulation using the “gformula” command in Stata [27] (example code given in Online Resource 1, part B). This method allows us to estimate mediation in the presence of intermediate confounding [31]. For the variable education we estimated the partial natural indirect effect by controlling for occupational noise or daily smoking. The effect of recurrent ear infections is an ordinary natural effect. The effects of occupational noise and smoking are complicated by the exposure-dependent confounder/mediator education. The natural effects were thus identified under the extra assumptions of no exposure-mediator interaction [32], or a linear effect of the exposure dependent confounder [33]. In order to choose between these two options, we tested for exposure-mediator interactions, exposure-intermediate interactions and nonlinearity of the intermediates [31].

We used a bootstrap with 1000 draws to estimate standard errors of natural indirect effects.

All models were controlled for age and sex. Due to sex differences in the cohort effect, analyses were also stratified on sex. To account for non-linearity, age was modelled as a restricted cubic spline with five knots. This created a better model fit than simpler models with age as a linear variable for all models tested (Likelihood-ratio test, *P*-value < 0.001).

#### Sensitivity analyses

We performed a simple sensitivity analyses of the assumption of no unmeasured mediator-outcome confounding by fitting structural equation models that allow for correlated error terms. As a measure of the strength of unmeasured mediator-outcome confounding we estimated the residual correlation, rho, by the method implemented in Stata by De Stavola to a setting with intermediate confounders [31]. A nonzero correlation can be interpreted as a measure of the strength of any unmeasured M-Y confounding that would imply an indirect effect of zero.

Subjects with missing data on any mediators (9%) were deleted list wise.

## Results

In HUNT2 Hearing, the subjects ranged in age from 20 to 101 years (median = 49.0, mean = 50.1, standard deviation = 16.9) with 53% women. In HUNT4 Hearing, the subjects ranged in age from 20 to 100 years (median = 54.0, mean = 53.2, standard deviation = 16.9) with 56% women. Table 1 shows that the prevalence of ear infections, occupational noise exposure, and daily smoking has decreased, whereas the prevalence of higher education has increased from HUNT2 to HUNT4.

**Table 1:** Distribution of risk-factors in HUNT2 (1996–1998) and HUNT4 (2017–2019), Norway

	Women		Men	
	HUNT2	HUNT4	HUNT2	HUNT4
	1996–1998	2017–2019	1996–1998	2017–2019
Age, years, mean (sd)	48.9 (16.6)	52.2 (16.7)	49.4 (16.2)	54.2 (16.7)
Education (%)				
Primary school	32%	13%	26%	12%
Secondary school	48%	43%	56%	56%
University < 4 years	18%	37%	13%	22%
University >=4 years	1%	6%	5%	9%
Recurrent ear infections (%)				
No	72%	80%	77%	85%
Maybe	5%	3%	6%	2%
Yes	23%	17%	17%	13%
Occupational noise exposure (%)				
No never	79%	86%	36%	58%
< 5 h per week	10%	4%	22%	12%
5–15 h per week	5%	6%	16%	16%
> 15 h per week	6%	4%	25%	15%
Daily smoking (%)				
No never	48%	56%	39%	56%
Previous	23%	35%	34%	38%
Current	29%	9%	27%	6%

The hearing thresholds improved from HUNT2 to HUNT4 by 2.8 dB at low frequencies (0.5–2 kHz) and 3.0 dB at high frequencies (3–6 kHz) (Cohort effect- Tables 2 and 3), with highest improvement in men.

**Table 2:** Mediation of the cohort difference in low frequency hearing thresholds (0.5, 1 and 2 kHz)

	Cohort effect^a^		Natural indirect effect		
	Estimate (dB)	95% CI	Estimate (dB)	95% CI	Mediated proportion
All:					
Total	−2.79	− 2.64, − 2.94	− 0.76^b^	− 0.85, − 0.68	0.27
Education			−0.28^c^	− 0.35, − 0.21	0.10
Recurrent ear infections			− 0.18^c^	−0.21, − 0.15	0.07
Occupational noise			−0.14^e^	− 0.18, − 0.10	0.05
Smoking			−0.14^e^	−0.21, − 0.08	0.05
Women:					
Total	−2.65	−2.45, − 2.84	−0.70^b^	− 0.81, − 0.59	0.26
Education			−0.38^c^	−0.48, − 0.27	0.14
Recurrent ear infections			−0.20^c^	−0.25, − 0.16	0.08
Occupational noise			−0.04^d^	−0.05, 0.02	0.01
Smoking			−0.05^d^	−0.09, − 0.02	0.02
Men:					
Total	−3.01	−2.78, −3.23	−0.91^b^	−1.06, − 0.77	0.30
Education			−0.22^c^	−0.31, − 0.13	0.09
Recurrent ear infections			−0.17^c^	−0.21, − 0.12	0.06
Occupational noise			−0.27^e^	−0.34, − 0.19	0.09
Smoking			−0.21^e^	− 0.33, − 0.08	0.07

All models were adjusted for age and sex

^a^ Difference in hearing thresholds between HUNT4 (2017–2018) and HUNT2 (1996–1998)

^b^ Estimated by fitting natural effect models in the R-package medflex using the imputation method including all exposure-mediation interaction terms

^c^ Estimated with gformula in Stata

^d^ Estimated with gformula in Stata with the assumption of no exposure mediation interaction as proposed by Robins and Greenland [32]

^e^ Estimated with gformula in Stata with the assumption of no exposure intermediate interaction together with only linear effects of the intermediate variable as proposed by Petersen et al. [33]

**Table 3:** Mediation of the cohort difference in high frequency hearing thresholds (3, 4 and 6 kHz)

	Cohort effect^a^		Natural indirect effect		
	Estimate (dB)	95% CI	Estimate (dB)	95% CI	Mediated proportion
All:					
Total	−2.97	− 2.76, − 3.18	−0.82^b^	− 0.94, − 0.70	0.28
Education			−0.12^c^	−0.22, − 0.02	0.04
Recurrent ear infections			−0.20^c^	−0.23, − 0.16	0.07
Occupational noise			−0.25^e^	−0.30, − 0.20	0.08
Smoking			−0.14^e^	−0.24, − 0.04	0.05
Women:					
Total	−1.25	−1.00, − 1.50	−0.68^b^	− 0.82, − 0.53	0.54
Education			−0.30^c^	−0.44, − 0.16	0.24
Recurrent ear infections			−0.21^c^	−0.26, − 0.16	0.17
Occupational noise			−0.06^d^	−0.08, − 0.04	0.05
Smoking			−0.08^d^	−0.13, − 0.04	0.07
Men:					
Total	−5.20	−4.85, −5.55	−1.39^b^	−1.61, − 1.18	0.27
Education			−0.35^c^	−0.47, − 0.22	0.07
Recurrent ear infections			−0.17^c^	−0.23, − 0.11	0.03
Occupational noise			−0.56^e^	−0.71, − 0.42	0.11
Smoking			−0.17^e^	−0.37, 0.03	0.03

All models were adjusted for age and sex

^a^ Difference in hearing thresholds between HUNT4 (2017–2018) and HUNT2 (1996–1998)

^b^ Estimated by fitting natural effect models in the R-package medflex using the imputation method including all exposure-mediation interaction terms

^c^ Estimated with gformula in Stata

^d^ Estimated with gformula in Stata with the assumption of no exposure mediation interaction as proposed by Robins and Greenland [32]

^e^ Estimated with gformula in Stata with the assumption of no exposure intermediate interaction together with only linear effects of the intermediate variable as proposed by Petersen et al. [33]

The joint natural indirect effects of all mediators were estimated to 0.8 dB (27%) and 0.8 dB (28%) respectively (Tables 2 and 3). The corresponding estimates found by traditional regression analyses assuming no interactions were 0.8 dB (29%) and 1.2 dB (40%) respectively.

Before detangling the mediation into specific effects, we selected between the identification assumptions of no exposure-mediator interaction and that of a linear effect of the exposure dependent confounder. Multivariate models of hearing thresholds including exposure-mediator interactions, exposure-intermediate interactions and nonlinearity term of the intermediate mediator/confounder revealed small exposure-mediator interactions and a small exposure-intermediate interaction at low frequencies for both women and men (see table in Online Resource 2). At high frequencies there was a negative interaction between cohort and occupational noise exposure and smoking, indicating reduced associations in the most recent cohort. An opposite interaction was found for recurrent ear infections. There was also an interaction between cohort and education, with less associations with education in the most recent cohort. Altogether, neither the assumption of no exposure mediation interaction nor the assumptions of no exposure intermediate interaction together with only linear associations of the intermediate variable was met, but suggested natural effects to be best identified under the extra assumptions of no exposure-mediator interaction for men, and using a linear effect of the exposure dependent confounder for women.

The specific natural indirect effects using the above assumptions indicated a mediation by all the tested mediators at both low (Table 2) and high (Table 3) frequencies and for both sexes. Recurrent ear infections was the strongest mediator in women, while occupational noise exposure was the most important mediator in men. Smoking also contributed to the improvement.

Sensitivity analysis estimated residual correlations, rho, of 0.12 and 0.08 for mediation of high frequency HT by occupational noise exposure and recurrent ear infections respectively.

Subjects with missing values of any of the mediators had slightly worse hearing than subjects participating in the analyses. This difference was estimated to 0.9 dB [95% CI 0.6–1.1] and 0.4 dB [95% CI 0.0–0.8] at low respectively high frequencies controlling for age and sex. The influence of list wise deletion on the cohort effect was however less than 1 %.

## Discussion

Our study showed that the better hearing in the more recent birth cohort of Norwegian adults to a large extent is attributed to secular trends in education, occupational noise exposure, recurrent ear infections and smoking. While occupational noise was the most important mediator in men, recurrent ear infections was most important in women.

The better hearing in the more recent born cohort is in agreement with other studies that suggest cohort improvements in hearing ability among adults [3–6]. However, our study provides the first evidence for that reductions in occupational noise exposure, ear infections and smoking has led to improved hearing at a population level.

Self-reported occupational noise exposure attenuated the cohort difference among men, especially at high frequencies. This agrees with a suggested reduction of noise-induced hearing loss in the industry in recent decades because of hearing conservation programs [34]. It is only within the past 40 years that serious efforts to reduce excessive noise at work sites have been initiated [35] and Norway implemented regulations to limit workers’ exposure to loud sounds with limits of 85 dB in 1982. While 65 years old persons in 2018 spent most of their working life after 1982, 65 years old in 1997 spent a major part of their working life before 1982 when hearing protector devices was less in use. There has been an uncertainty about the effectiveness of hearing loss prevention interventions, and a recent review reported a lack of evidence for that preventive measures reduce the risk of occupational hearing loss [36]. As such, our study adds important findings on this topic.

A history of recurrent ear infections has been associated with poorer hearing thresholds [37]. Our study showed a reduced prevalence of self-reported recurrent ear infections from HUNT2 to HUNT4. This may be a result of improved living standard, health care and hygiene, and the introduction of antibiotics [38]. The reduction attenuated the cohort difference at low and high frequencies for both sexes. Our result contradicts the finding by Zhan et al. 2011, who used data from studies conducted in Beaver Dam, Wisconsin [22]. The authors found the prevalence of a history of ear infection to have increased from 1993 to 1995 to 2005–2008 and increasing the birth cohort effect. We have no explanation for this discrepancy, but one difference is that we asked for recurrent ear infections instead of single episodes of ear infections, and the Beaver Dam studies were restricted to the population of adults above 45 years of age and a shorter time-span of 10 years [5].

Despite a large decline in smoking consumption, smoking explained only a minor part of the change in hearing. We believe that this finding is plausible, since smoking has been associated with hearing with modest effect sizes [19, 39–41].

Our study showed that increased educational attainment explained some of the cohort difference, which agrees with the study by Zhan et al. 2011 [22]. Socioeconomic status (SES) is usually measured by education, income, or occupation, and a relationship with hearing loss have been shown both in cross sectional studies [21, 42–44] and prospective studies [45]. It seems probable that the association between SES and hearing loss is mediated by known risk factors, such as noise exposure, smoking, diabetes, and hypertension [46]. Because we had statistical power to detangle specific indirect effects, we could show that the other risk factors contributed as much as education to the attenuation of the cohort effect when treating education as an intermediate confounder.

A recent follow-up of the studies in Beaver Dam assessed generational decreases in 5-year hearing loss incidence and 10-year cumulative hearing loss incidence. The study failed to ascribe the decreases to changes in any of the measured known risk factors for hearing loss including work related noise, smoking status and educational level [23]. They therefore speculated about whether other as-yet unidentified factors were responsible for the changes. The study mainly showed a higher risk of hearing loss in the oldest generation born between 1901 and 1924. Again we cannot explain with certainty the discrepancy and lack of significant contributions of the known risk factors in the Beaver Dam study, however it could be related to methodological differences such as different outcome measures (incidence and 10-year change in hearing threshold), different measures of the risk factors, shorter time-span of 10 years, less overlap between age and generation, and fewer participants. There might also be differences in the environmental changes between USA and Norway in terms of development of hearing conservation programs and improvement in general health.

Reports from the US National Health and Nutrition Examination showed improvement of hearing between 1959 and 1962 and 1999–2004 and between 1999 and 2004 and 2011–2012 for adults aged 25 to 64 years and 20 to 69 years respectively [3, 4]. As the first report was over a longer period with the oldest subjects being one generation older than the oldest participants in our study, they spanned over the period in which hearing conservation programs was introduced. But improvements in reducing infections and malnutrition at the beginning of the century may have had an impact on the oldest subjects. The explanation for a continuing trend presented in the later report may be in line with ours.

Our results may be compared with a Swedish study that showed improved hearing in 70-year-old subjects born in 1944 compared with those born in 1922 and 1901–1907 [6]. Although we included the whole adult population, the contributions to our improvements were dominated by 65–75 years old male subjects in whom the improvement was largest [7]. Our 70 years old subjects were born in about 1927 and 1948 in our respectively cohorts and therefore comparable with the Swedish birth cohorts. It is therefore likely that a reduction in occupational noise exposure, ear infections and smoking also explained some of the improvements found in the Swedish study, as Sweden and Norway have had a comparable development of hearing conservation programs and improvement in general health. The authors also pointed at reduction in occupational noise as one possible explanation [6].

Other factors may also have contributed to better hearing in the present study. For example, focus on hearing protection and improved regulation in hunting and sports-shooting may have had an effect. There has also been an improvement in life-style factors other than smoking that we did not measure, such as lack of physical activity/exercise. The improvement may as well be explained by factors related to prenatal and early childhood development such as reduction in infections due to introduction of antibiotics and vaccination, head traumas or use of ototoxic drugs. On the contrary, there are factors that have worsened, such as diabetes, body mass index, and exposure to music through earphones.

### Strengths and weaknesses

The major strength of our study is the large population-based design with cohorts separated 20 years apart, and the use of standardized audiometric procedure and contemporary mediation approach.

There are also limitations. With measures at only two time points we cannot derive the point at when hearing started to improve, or how it changed. Using the data to forecast future hearing status of the population is therefore limited. Second, we cannot fully reject possible influences of selection bias. As in most observational studies, our recent study wave experienced falling response rates and perhaps a healthier population. The observed birth cohort difference in hearing has previously been shown to be little effected by bias due to nonparticipation differences between the two study waves [7], nor was it much influenced by the present use of complete-case analyze excluding missing mediators. Third, the conclusions may not be generalizable to other populations with different exposure pattern. Fourth, the mediation analyses assume no unmeasured confounding between exposure and the outcome, between mediator and the outcome or between exposure and the mediator. In addition, for estimates to be interpreted as natural direct and indirect effects, there should be no mediator-outcome confounder that is itself affected by the exposure (or other restrictions) [47]. With fixed exposure there was no exposure-outcome or exposure-mediator confounding. Results of the sensitivity analysis indicated residual correlation between the mediator and outcome to be at least 0.12 in order to completely remove the path mediated by occupational noise and 0.08 to remove the path mediated by recurrent ear infections. Although these are not large correlations, we are not aware of any such factors that are strongly related to hearing threshold in the general population, other than genetics that cannot explain such fast secular changes.

## Conclusion

This study showed that increased education, less occupational noise exposure, ear infections and smoking contribute substantially to improved hearing in Norway the last two decades. Strategies for prevention of these risk factors seem to be successful and might have a strong impact for reducing hearing loss at a population level.

## Supplementary Information

**Additional file 1.**

**Additional file 2.**

## Data Availability

The datasets generated and/or analyzed during the current study are not publicly available due to Norwegian legal restrictions and the current ethical approval for the study, but descriptive data in table form are available from the corresponding author on reasonable request.

## Abbreviations

HUNT: The Trøndelag Health Study
DAG: Directed acyclic graph (DAG)
HT: Hearing threshold
