# Supplementary material for: Explaining better hearing in Norway: a comparison of two cohorts 20 years apart - the HUNT study
Source: BMC Public Health. 2021 Jan 28;21:242. doi: 10.1186/s12889-021-10301-1 (PMC7844976; doi:10.1186/s12889-021-10301-1)
Supplement: Supplementary file 1 — Additional file 1. [file 12889_2021_10301_MOESM1_ESM.docx]

Online Resource 1.

**A. Mediation analysis fitting the joint mediated effect using imputation-based natural effect models of the R package “medflex”**

**l**ibrary(medflex)

# Covariates - ages1 ages2 ages3 ages4 sex (ages1-ages2 are splines of age)

# y – low frequency or high frequency hearing threshold

# x – cohort

# e- education, w-occupational noise, r – recurrent ear infection, s-smoking

impFit <- glm(y ~ x+ e + r + w + s + x:e + x:w + x:r + x:s + ages1 + ages2 + ages3 + ages 4 + sex,

family = gaussian, data = mydata)

expData <- neImpute(impFit, nMet=4)

neMod <- neModel(y ~ x0x1 + ages1 + ages2 + ages3 + ages4 + sex, family = gaussian,

expData= expData, se= “bootstrap”)

effedecomp <- neEffdecomp(neMod)

**B. Mediation analysis with intermediate confounding. G-computation by gformula in Stata**

// Covariates - ages1 ages2 ages3 ages4 sex (ages1-ages2 are splines of age)

global c ages1 ages2 ages3 ages4 sex

// y - low frequency or high frequency hearing threshold

// x – cohort

// m – mediator (occupational noise or smoking)

// l – intermediate (education)

*** Model 1 Robins & Greenland

gformula x l m y xm xl l2 $c, ///

mediation outcome(y) ///

eq(l:x $c, m: x l xl $c, ///

y:x l m l l2 xl $c) com(m:regress, y:regress, l:regress) ///

exposure(x) mediator(m) control(m:0) obe ///

post_confs(l) base_confs($c) derived(xl l2) ///

derrules(xl:x*l, l2:l*l) ///

sam(1000) seed(79)

scalar nie=r(nie) // natural indirect effect

scalar ll=nie-1.96*r(se_nie)

scalar ul=nie+1.96*r(se_nie)

scalar mp=r(nie)/r(tce) // Mediated proportion

*** Model 2 Petersen et al

gformula x l m y xm xl $c, ///

mediation outcome(y) ///

eq(l: x $c, m: x l xl $c, ///

y: x l m xm $c) com(m:regress, y:regress, l:regress) ///

exposure(x) mediator(m) control(m:0) obe ///

post_confs(l) base_confs($c) derived(xm xl) ///

derrules(xm:x*m, xl:x*l) ///

sam(1000) seed(79)

scalar nie=r(nie) // natural indirect effect

scalar ll=nie-1.96*r(se_nie)

scalar ul=nie+1.96*r(se_nie)

scalar mp=r(nie)/r(tce) // Mediated proportion
