# Supplementary material for: Explaining better hearing in Norway: a comparison of two cohorts 20 years apart - the HUNT study
Source: BMC Public Health. 2021 Jan 28;21:242. doi: 10.1186/s12889-021-10301-1 (PMC7844976; doi:10.1186/s12889-021-10301-1)
Supplement: Supplementary file 2 — Additional file 2. [file 12889_2021_10301_MOESM2_ESM.docx]

| **Online Recourse 2.**  Estimated coefficients from multivariable regression models including interactions. HUNT2 (1996-1998) and HUNT4 (2017-2019), Norway | | | | | | | |
| --- | --- | --- | --- | --- | --- | --- | --- |
|  |  | Low frequency hearing thresholds (0.5, 1 and 2 kHz) | | | High frequency hearing thresholds (3, 4 and 6 kHz) | | |
|  | Variable | Point Estimate (dB) | | 95% CI | Point Estimate (dB) | | 95% CI |
| All: | Education | -2.35 |  | -2.75, -1.95 | -2.26 |  | -2.85, -1.68 |
|  | Recurrent ear infections | 1.31 |  | 1.20, 1.41 | 1.18 |  | 1.03, 1.34 |
|  | Occupational noise exposure | 0.72 |  | 0.63, 0.80 | 1.84 |  | 1.72, 1.96 |
|  | Smoking | 0.27 |  | 0.16, 0.37 | 0.88 |  | 0.73, 1.03 |
|  | Education squared | 0.31 |  | 0.22, 0.40 | 0.26 |  | 0.13, 0.39 |
|  | cohort x education | 0.20 |  | 0.00, 0.40 | 0.53 |  | 0.24, 0.82 |
|  | cohort x recurrent ear infections | 0.24 |  | 0.05, 0.43 | 0.44 |  | 0.16, 0.71 |
|  | cohort x noise exposure | -0.12 |  | -0.26, 0.02 | -0.77 | ^b^ | -0.97, -0.56 |
|  | cohort x smoking | 0.16 |  | -0.06, 0.37 | -0.45 | ^b^ | -0.76, -0.14 |
| Women: | Education | -2.04 |  | -2.61, -1.48 | -2.84 |  | -3.56, -2.11 |
|  | Recurrent ear infections | 1.30 |  | 1.16, 1.44 | 1.39 |  | 1.22, 1.57 |
|  | Occupational noise exposure | 0.29 |  | 0.15, 0.44 | 0.56 |  | 0.38, 0.75 |
|  | Smoking | 0.06 |  | -0.09, 0.20 | 0.15 |  | -0.03, 0.33 |
|  | Education squared | 0.23 |  | 0.09, 0.36 | 0.38 | ^a^ | 0.21, 0.55 |
|  | cohort x education | 0.27 |  | -0.01, 0.54 | 0.47 | ^a^ | 0.13, 0.82 |
|  | cohort x recurrent ear infections | 0.23 |  | -0.02, 0.47 | 0.19 |  | -0.12, 0.50 |
|  | cohort x noise exposure | 0.17 |  | -0.07, 0.42 | 0.10 |  | -0.21, 0.42 |
|  | cohort x smoking | 0.29 |  | 0.01, 0.57 | 0.28 |  | -0.08, 0.63 |
| Men: | Education | -2.74 |  | -3.33, -2.15 | -2.59 |  | -3.50, -1.67 |
|  | Recurrent ear infections | 1.33 |  | 1.17, 1.50 | 1.01 |  | 0.75, 1.26 |
|  | Occupational noise exposure | 0.92 |  | 0.81, 1.03 | 2.09 |  | 1.92, 2.26 |
|  | Smoking | 0.39 |  | 0.23, 0.56 | 0.71 |  | 0.46, 0.96 |
|  | Education squared | 0.41 |  | 0.29, 0.54 | 0.21 |  | 0.01, 0.41 |
|  | cohort x education | 0.01 |  | -0.29, 0.31 | 0.34 |  | -0.12, 0.81 |
|  | cohort x recurrent ear infections | 0.26 |  | -0.05, 0.57 | 0.58 |  | 0.10, 1.06 |
|  | cohort x noise exposure | -0.30 |  | -0.49, -0.10 | -0.80 | ^b^ | -1.10, -0.50 |
|  | cohort x smoking | 0.07 |  | -0.27, 0.41 | -0.34 | ^b^ | -0.86, 0.18 |
| ^a^ Indication for the assumption of no exposure mediation interaction as proposed by Robins and Greenland [1]. | | | | | | | |
| ^b^ Indication for the assumption of no exposure intermediate interaction together with only linear effects of the intermediate variable as proposed by Petersen et al.[2]. | | | | | | | |

1. Robins JM, Greenland S. Identifiability and exchangeability for direct and indirect effects. Epidemiology. 1992;3(2):143-55. doi:10.1097/00001648-199203000-00013.

2. Petersen ML, Sinisi SE, van der Laan MJ. Estimation of direct causal effects. Epidemiology. 2006;17(3):276-84. doi:10.1097/01.ede.0000208475.99429.2d.
